# Quality control of bedside DR in neonatal chest radiography using a chest stabilization device and its clinical application

**DOI:** 10.1186/s12887-020-02228-0

**Published:** 2020-07-14

**Authors:** Xinqi Zhang, Yinfeng Zhang

**Affiliations:** Department of Radiology, Second Hospital of Hebei Medical University, No 215 West Heping Road, Xinhua District, Shijiazhuang, 050000 Hebei China

## Abstract

**Background:**

To develop a method for movement control during radiation exposure and to improve image quality of bedside thoracic DR in neonates.

**Methods:**

Total 60 cases of neonates’ thoracic DR X-ray images, which were taken before and after neonates’ movement control, were compared and analyzed. X-ray exposure was set at 47 kV/1.4 mAs for all films that were taken without movement control, while various exposure conditions were used based on the neonate’s body weight when the neonate’s movement was controlled.

**Results:**

The radiation dose of X-ray exposure was significantly lower after neonates’ movement control (7.32 ± 0.20 μGy) than that before the movement control (24.20 ± 0.82 μGy, *P* <  0.05), and it was decreased most dramatically in the neonates with lowest body weight (70%). After neonates’ movement control, image quality was significantly improved (44 cases out of 60, 73.3%) compared to that before movement control (only 5 out 60, 8.3%, *P* <  0.05). There was no significant difference in the score of image background noise before and after movement control (*P* <  0.05).

**Conclusion:**

Movement control with simple device could not only significantly improve the image quality, but also remarkably reduce radiation exposure dose.

## Background

Neonates, especially preterm neonates, often require bedside DR chest X-ray examination [1]. DR X-ray has been widely used in the neonates ICU or ward in that it is portable, easy to perform, and provide important image evidence for the diagnosis and differential diagnosis of lung problems in the neonates [2]. However, due to neonate’s movement during X-ray exposure, image quality of the neonates’ chest DR X-ray is often affected. In addition, portable bedside X-ray device has low capacity of image processing function, which may also contribute to the result of low quality image [3]. Poor image leads to taking multiple X-ray exposure, which results in over-dose radiation exposure for the neonates. Aim of the current study was, therefore, to develop a simple method to control neonate’s movement during the X-ray exposure in order to obtain better quality image, and by which to reduce radiation exposure for the neonates.

## Methods

### Neonate inclusion and general characteristics

Total 120 neonates, who met the following inclusion criteria, were enrolled into this study. Inclusion criteria: 1). Body weight < 4 kg; 2). Neonates without heart failure; 3). Neonates who were not on ECG monitoring. These 120 neonates were grouped into 8 groups (15 cases each group) by their body weight and neonates’ movement control as following. Before the movement control: Group A1, body weight ranged 1.2 ~ 1.4 kg; Group B1, body weight ranged 1.5 ~ 2.2 kg; Group C1, body weight ranged 2.3 ~ 3 kg; and Group D1, body weight > 3 kg. After the movement control, they were grouped as Group A2: body weight ranged 1.2 ~ 1.4 kg; Group B2: body weight ranged 1.5 ~ 2.2 kg; Group C2: body weight ranged 2.3 ~ 3 kg; and Group D2: body weight > 3 kg.

### Equipment

DRX-revolution mobile X-ray system (USA) was used. Maximum current: 320 mA; amorphous silicon detector; with 3.6 mp/mm resolution; Fuji dry laser digital printer.

### Radiological examination procedure

Procedure without neonate’s movement control: the neonates were on supine position and placed on the detection board with sand bags on their legs. Exposure area was 20 × 20 cm and non-exposure area were covered with protection. Center-line of the exposure was at top 1/3 of sternum [4]; exposure distance: 90 ~ 110 cm; exposure apparatus: 47 kV/0.5mAs. X-ray images were transferred to PACS system after processing at the station.

**Fig. 1:**
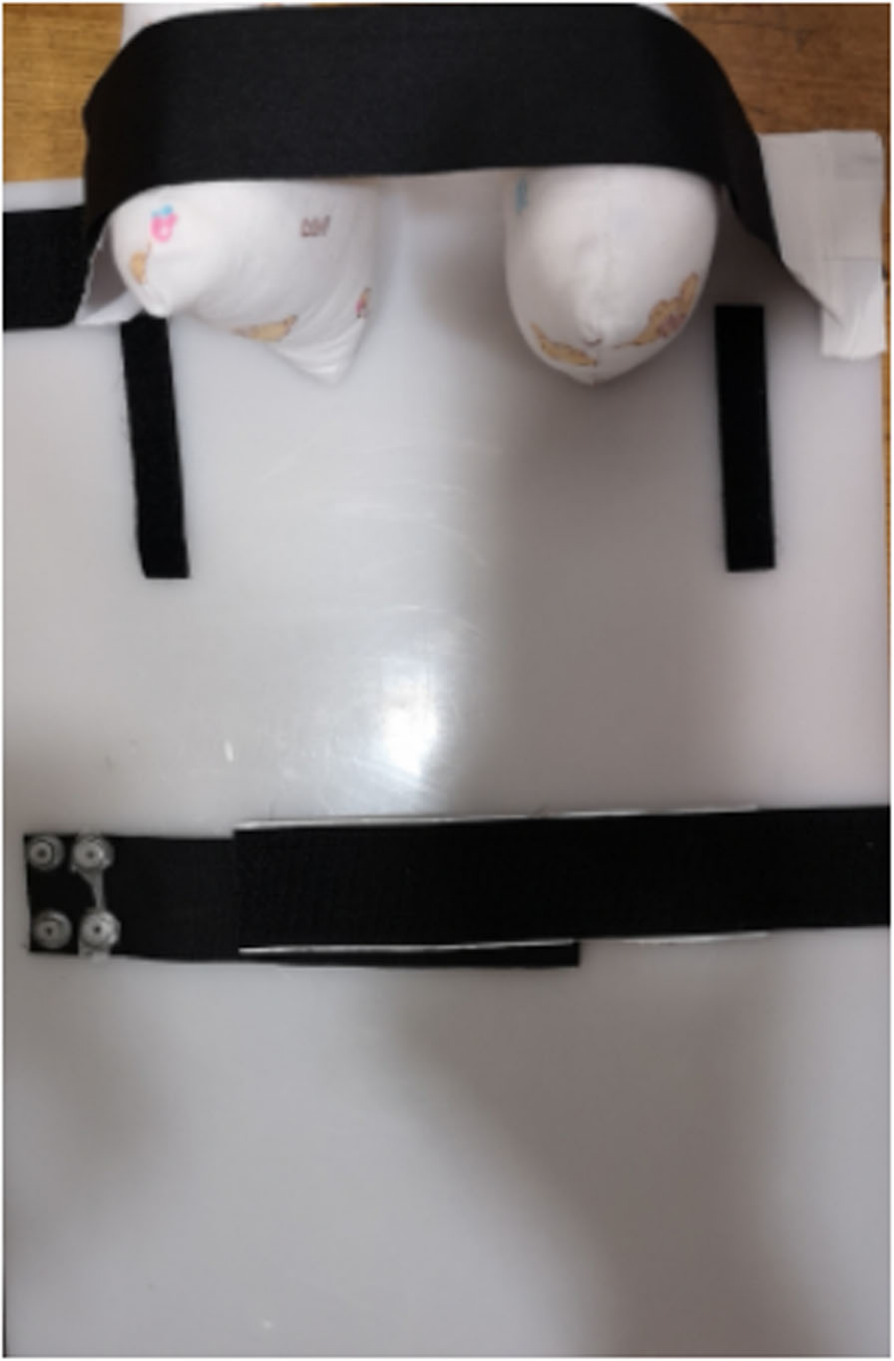
Neonate chest stabilization device. Belt at lower part of the PVC board was used to stabilize neonate’s abdomen, and belt at upper part of the PVC board was used to stabilize neonate’s head. Sandbag was used to stabilize neonate’s arms

Procedure with neonates’ movement control: a chest fixation device was used in order to control movement of the neonates. As shown in Fig. 1, this device was consisted of X-ray permeable PVC board, belts, sand bags and cotton bags. The PVC board was placed in the center of detector, and the neonate’s head, legs, arms, and abdomen were controlled with the sand bags, cotton bags and belts. Exposure apparatus was determined based on the neonate’s body weight as summarized in Table 1. Exposure area was adjusted by the size of the neonate’s chest size. Other settings were same as before the movement control.

**Table 1:** Film exposure conditions before and after movement control (*n* = 15)

Body weight	Before (Kv/mAs)	After (Kv/mAs)
1.1 ~ 1.4 kg	47 / 1.4	48 / 0.5
1.5 ~ 2.2 kg	47 / 1.4	52 / 0.5
2.3 ~ 3.0 kg	47 / 1.4	55 / 0.5
> 3 kg	47 / 1.4	61 / 0.5

### Radiation dosage

A radiation detector was used to measure the radiation dose received by each neonate. The radiation detector was composed by 3 TLD wrapped in the black paper. This radiation detector was placed at exposure center-line, which was sent to the Radiation Institute of Hebei Province to measure the radiation dose [5].

### Image quality evaluation

The image quality was evaluated and scored by two experienced Radiologists. If the two Radiologists had different opinion on the image quality, they discussed together to achieve an agreement on the scoring. Total 10 points scoring was used as following: 1). Symmetrical chest image (1 point); 2). Centralized mediastinum (2 points); 3). Clear lung texture, heart image, and diaphragm (1 point); 4). Clear and sharp costophrenic angles (1 point); 5). Appropriate contrast and clean image (1 point); 6). Appropriate density (1 point); 7). Low background or noise (1 point); 8). Appropriate exposure area (1 point); 9). Both lungs were included in images (1 point). Score of 10 was considered as excellent image and < 6 was considered as disqualified image. Image background or noise was evaluated the 3 points system as following: images with very low background or noise, and met the requirement for diagnosis was scored as 3; images with mild background or noise, but could be used for diagnosis was scored as 2; images with moderate background or noise, and could not be used for diagnosis was scored as 1; images with significant background or noise and could not be used for diagnosis was scored as 0 [6].

### Statistical analysis

SSPS 20.0 software was used for the statistical analysis. Student t test was used for the comparison of radiation dose between the groups. Wilcoxon rank-sum test was used for the comparison of quality score evaluation. *P* <  0.05 was considered as significant.

## Results

### General characteristics of the neonates

Average age of the 4 groups before the movement control was 6 h ~ 20 day; body weight ranged 1.2 ~ 4 kg; 32 boys and 28 girls; chest X-ray found lung infection (6 cases), ARDS (4 cases), lung hyaline membrane disease (10 cases), PICC and trachea intubation (12 cases), and normal lungs (28 cases).

Average age of the 4 groups after the movement control was 6 h ~ 18 day; body weight ranged 1.3 ~ 3.8 kg; 29 boys and 31 girls; chest X-ray found lung infection (8 cases), ARDS (6 cases), lung hyaline membrane disease (14 cases), trachea intubation (12 cases), and normal lungs (20 cases). There were no significant differences between the groups before and after neonates’ movement control in the ratio of gender, body weight, or age (*P* > 0.05).

### Comparison of radiation exposure dose before and after neonates’ movement control

As shown in Table 2, radiation exposure dose was significantly lower after the movement control in all 4 different body weight groups compared to that before the movement control (24.20 ± 0.82 versus 7.32 ± 0.20 μGy, *P* <  0.01 in the group A; 24.51 ± 0.78 versus 10.56 ± 0.59 μGy, *P* <  0.01 in the group B; 24.54 ± 0.64 versus 12.57 ± 0.37 μGy, *P* <  0.01 in the group C; 24.52 ± 0.04 versus 14.59 ± 1.08 μGy, *P* <  0.01 in the group D).

**Table 2:** Comparison of radiation dose before and after movement control (*n* = 60, μGy)

Group	Before	After	*P*
A1/A2	24.20 ± 0.82	7.32 ± 0.20	< 0.001
B1/B2	24.51 ± 0.78	10.56 ± 0.59	< 0.001
C1/C2	24.54 ± 0.64	12.57 ± 0.37	< 0.001
D1/D2	24.52 ± 0.04	15.59 ± 1.08	< 0.001

### Image quality evaluation

**Fig. 2:**
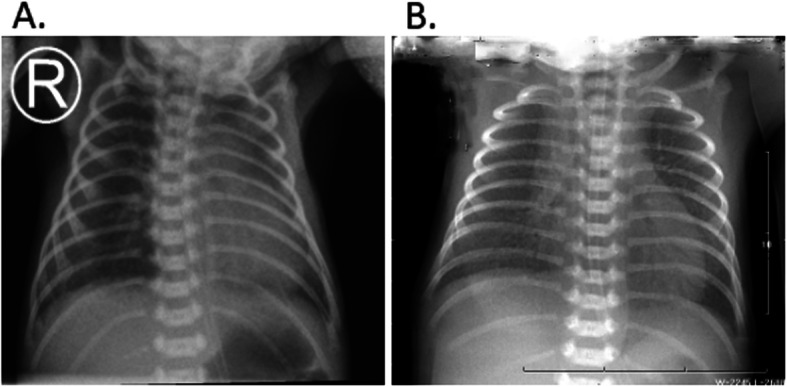
Representative images before and after neonates’ movement control. Panel **a**: Representative image before the movement control. A male neonate, one day after born, and 1.75 kg body weight. Asymmetric thoracic image, twisted image, mediastinum moved towards left, and unclear lung texture, scored 6. Panel **b**: Representative image after the movement control. A female neonate, one day after born, and 1.55 kg body weight. Symmetric thoracic image, centered mediastinum, clear lung texture, scored 10

As shown in Table 3, image quality was significantly improved after the movement control. The number of high quality image after the movement control was 44 out of 60 images (zero poor quality image), while it was 5 out 60 images before the movement control (8 images were very poor and unusable), and there was significant difference before and after the movement control (*P* < 0.05, Table 3 and Fig. 2). Before the movement control, 42 images were asymmetric thoracic images, 38 images were not centered mediastinum image, 14 images were unclear lung texture caused by diaphragm movement, 4 images were incomplete lung field, and 8 images were poor contrast image. In contrast, after the movement control, none of the images were asymmetric thoracic image or non-centered mediastinum, only 2 images were unclear lung texture caused by diaphragm movement, zero incomplete lung field image, and 4 images with poor contrast.

**Table 3:** Comparison of image quality before and after movement control (n = 60)

Score (point)	4	5	6	7	8	9	10	z	*P*
Before	2	6	15	14	5	13	5		
After	0	0	0	0	2	14	44	8098	0.000

There was no significant difference in image noise before and after the movement control (Table 4, *P* > 0.05).

**Table 4:** Comparison of image noise before and after movement control (n = 60)

Group	1	2	3	z	*P*
Before	4	4	52		
After	2	10	48	0.596	0.551

## Discussion

Portable bedside DR X-ray is widely applied in clinic, especially, for the diagnosis of lung hyaline membrane disease in preterm neonates [7]. However, neonates were often over-exposed to radiation, which was harmful to neonates and might cause tumorous change [8, 9], especially in the preterm neonates, in that cell division in neonates is faster than that in adult [10]. Therefore, it is crucial to reduce radiation exposure for the neonates. The current study was designed to compare the radiation exposure in the neonates who had portable bedside DR X-ray examination before and after movement control.

In clinical practice, The DR chest X-ray images of neonates often turned out to be asymmetric, twisted or unclear images due to the uncontrolled movement of the neonates. In the current study, therefore, we have used PVC board, belts, and sand bags to control the neonate’s movement during DR chest X-ray exposure, by which, quality of bedside DR chest X-ray image was dramatically improved. In addition, application of this simple device to control body movement could not only improved image quality, but also significantly reduced radiation exposure to the neonates in that repeated X-ray examination was avoided after movement control.

Body weight is one of the important factors associated with neonate’s growth. Neonates with heavier body weight have thicker chest wall and thus, higher voltage is required for X-ray penetration. Therefore, in the current study, voltage was adjusted based on the neonates’ body weight. Specifically, 61 kV was used for neonates with body weight of 6 kg or higher, and 48 kV was used for neonates with body weight of 1.4 kg or less. Through this adjustment, background noise was not significantly increased when the voltage was reduced in the lighter neonates. In addition, adjustment of the X-ray exposure based on the body weight resulted in significant reduction of radiation exposure (up to 70% reduction in the neonates with lowest body weight), while background noise was not significantly increased. These results suggested that X-ray exposure could be adjusted by neonate’s body weight. In the current study, there were no significant differences in the quality of images after movement control among the four different weight groups (data not shown).

It has been reported that several methods were used to control neonates’ movement during chest X-ray filming [2, 7]. In this regard, sand bags and bandages have been used to control the movement of neonates’ head and extremities. However, movement control with sand bag or bandage alone was not stable and the images of chest and abdominal X-ray were twisted or the interested field was covered by the sand bag due to neonate’s movement [11]. In the current study, of the 42 images taken without movement control, 11 images were twisted, 31 images were asymmetric due to head movement. Therefore, we used PVC board, belts and sand bags to control movement of neonate’s head, body and extremities. After movement control with these simple stuffs, images of bedside chest X-ray were significantly improved. That is, after neonates’ movement control, all of the chest DR images were symmetric, non-twisted, and met the criteria for disease diagnosis differential diagnosis; only 2 images were unclear; 44 out of 60 images (73%) were in excellent quality. Findings of this study demonstrated that this simple device could be used for effectively controlling neonate’s movement during DR X-ray exposure, and by which, it significantly improved quality of the images and reduced background noise of the images. In addition, movement control led to significant reduction of X-ray exposure intensity (declined from1.4 mAs to 0.5 mAs) or exposure length (3 times reduction) without affecting the quality of the images.

The device used in the current study was simple to use, inexpensive, portable from room to room, and required a very brief training for the user. However, there were following limitations of this study. 1). The device is not suitable for a neonate in sedated. 2). Neonates with ECG monitoring were excluded from this study. 3). Neonates with heart failure were excluded from this study.

## Conclusion

In conclusion, simple device of PVC board, belts and sand bags could be used for movement control of neonates. Control of neonates’ movement during DR X-ray imaging could not only significantly improve the quality of the images, but also reduced X-ray exposure intensity and time.

## Data Availability

The datasets generated and analyzed during the current study are available from the corresponding author on reasonable request.
